# Soluble Receptors Affecting Stroke Outcomes: Potential Biomarkers and Therapeutic Tools

**DOI:** 10.3390/ijms22031108

**Published:** 2021-01-23

**Authors:** Ayon Bhattacharya, Rani Ashouri, Madison Fangman, Alexandra Mazur, Timothy Garett, Sylvain Doré

**Affiliations:** 1 Department of Anesthesiology, University of Florida College of Medicine, Gainesville, FL 32610, USA; ayon2385@ufl.edu (A.B.); ashourir15@ufl.edu (R.A.); maddiefang@ufl.edu (M.F.); alexandrammazur@ufl.edu (A.M.); 2 Departments of Pathology, Immunology and Laboratory Medicine, University of Florida, Gainesville, FL 32610, USA; tgarrett@ufl.edu; 3 Departments of Neurology, Psychiatry, Pharmaceutics, and Neuroscience, Center for Translational Research in Neurodegenerative Disease, McKnight Brain Institute, University of Florida, Gainesville, FL 32610, USA

**Keywords:** ADAM, sCD36, sCD163, sLRP1, decoy receptors, neuroinflammation, ischemia, hemorrhage, stroke

## Abstract

Soluble receptors are widely understood to be freestanding moieties formed via cleavage from their membrane-bound counterparts. They have unique structures, are found among various receptor families, and have intriguing mechanisms of generation and release. Soluble receptors’ ability to exhibit pleiotropic action by receptor modulation or by exhibiting a dual role in cytoprotection and neuroinflammation is concentration dependent and has continually mystified researchers. Here, we have compiled findings from preclinical and clinical studies to provide insights into the role of soluble/decoy receptors, focusing on the soluble cluster of differentiation 36, the soluble cluster of differentiation 163, and soluble lipoprotein-related protein 1 (sCD36, sCD163, and sLRP1, respectively) and the functions they could likely serve in the management of stroke, as they would notably regulate the bioavailability of the hemoglobin and heme after red blood cell lysis. The key roles that these soluble receptors play in inflammation, oxidative stress, and the related pharmacotherapeutic potential in improving stroke outcomes are described. The precise pleiotropic physiological functions of soluble receptors remain unclear, and further scientific investigation/validation is required to establish their respective role in diagnosis and therapy.

## 1. Introduction

In ischemic stroke, a pro-thrombotic and embolic milieu exacerbated by inflammatory conditions becomes a disposition toward cerebrovascular blockage and ischemia. After an ischemic event, a cascade of secondary damage ensues. This cascade includes bioenergetic failure, ecotoxicity, microvascular injury, oxidative stress, and inflammation. Of particular interest is the resulting breakdown of the blood–brain barrier (BBB), which has been noted to be mediated by cytokines, chemokines, matrix metallopeptidases, and other inflammation and oxidative stress mediators [1]. Concurrently, hemorrhagic stroke is postulated to lead to the rapid deterioration of the BBB, likely through the increase in hemoglobin and heme after red blood cell (RBC) lysis [2]. This breakdown of the BBB thus leads to an influx of immune cells destructive to proper neurologic function. Therefore, due to the role that soluble receptors play in modulating and marking inflammatory and oxidative pathway function, we aim to better define their activity as possible therapeutic targets and biomarkers for stroke clinical treatment.

Soluble receptors are the derivatives of membrane-bound receptors and are produced under a wide variety of conditions. While these receptors are most prevalent in the tumor necrosis factor (TNF; Fas ligands) and hematopoietic receptor superfamilies, they can also be found in numerous other receptor groups, such as immunoglobulin, tyrosine kinase, adhesion molecules, and growth hormone [3]. The soluble receptors play a significant role in maintaining a dynamic equilibrium among themselves, the membrane receptor, and the ligand, with each component interacting in highly specific ways to regulate many downstream processes.

**Figure 1:**
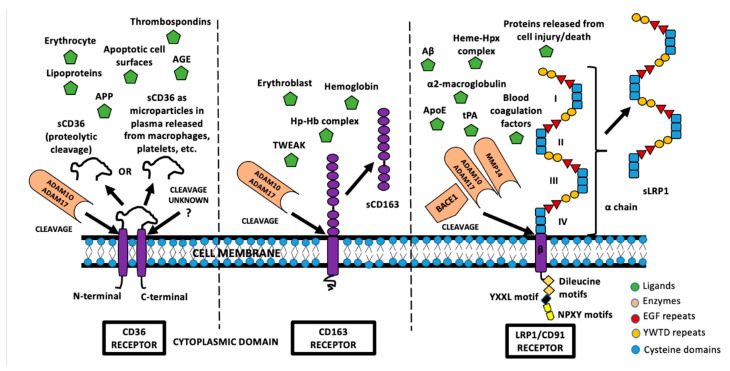
An illustration depicting the (patho)physiological release of soluble receptors from membrane receptors and the various ligands that they can control their bioavailability. Note that, for the CD36 receptor, two types of soluble forms exist, one of which the mechanism of cleavage remains unknown. In addition, the TNF-related weak inducer of apoptosis (TWEAK) is also known as Fn14. Other abbreviations denoted above are advanced glycation end-product (AGE), amyloid precursor protein (APP), haptoglobin–hemoglobin (Hp–Hb) complex, heme–hemopexin (Hpx) complex, apolipoprotein (ApoE), and tissue plasminogen activator (tPA).

The generation and release of soluble receptors occur through a multitude of mechanisms throughout the body. Soluble receptors can be generated from alternative splicing of mRNA transcripts, as by-products of protein synthesis, or via proteolytic cleavage, possibly as part of membrane-bound receptor degradation. The differential splicing of mRNA transcripts led to the generation of soluble protein molecules with the deletion of the transmembrane domain. To highlight an example of soluble receptor generation, studies have shown that CD36 receptors could be an unbound protein or microparticle from other cells rather than a proteolytic product [4]. An illustration of the generation of soluble receptors is provided in Figure 1, alongside a few key ligands associated with each of the selected receptors.

It should be noted that generation and release mechanisms are not mutually exclusive. The exosomal release of receptors in the extracellular space is another mechanism for soluble receptor generation. The release mechanism most commonly used is referred to as ectodomain shedding, where there are limited proteolytic activities in the membranous receptor’s extracellular region [5]. Proteolytic shedding primarily occurs constitutively, although it can also be induced with ligand binding or stimulation by chemicals such as phorbol 12-myristate 13-acetate and ionomycin [6]. A zinc-dependent metzincin family of metalloproteinase, known as ADAM (a disintegrin and metalloproteinase domain-containing protein 10) is responsible for the proteolytic shedding of membrane receptors, as seen in the generation of sCD163 and sLRP1 [7,8,9]. Although there are various examples, the primary enzymes responsible for shedding are ADAM10 and ADAM17; ADAM17 is also referred to as the TNF-converting enzyme (TACE) [10,11].

Soluble receptors’ wide versatility is continually revealed and can range from biomarkers of disease pathology to regulation of membrane receptor function. For example, studies are currently looking into the roles sLRP1 and soluble receptor for advanced glycation end-product (sRAGE) play in Alzheimer’s disease (AD). LRP1 promotes the efflux of Aβ from the brain, which crosses the BBB into the blood circulation, whereas RAGE regulates the influx of β-amyloid (Aβ) from blood circulation into the brain, exacerbating the pathology of AD [12]. Thus, knowledge of membrane receptors such as RAGE and LRP1 has led us to explore their corresponding soluble receptors. Moreover, the role of genetic polymorphisms in soluble receptors is currently being studied due to the fact a single nucleotide polymorphism of ADAM10 has been associated with an increased risk of AD [13]. This indicates the putative protective benefits that soluble receptors may play in disease pathology.

**Figure 2:**
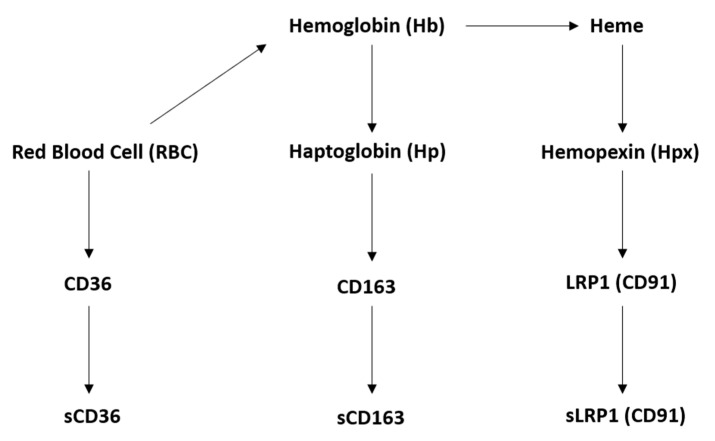
Molecular association of red blood cells, Hp–Hb, and the Hpx–Heme complexes with its respective receptor and corresponding decoy receptor.

Soluble receptors also present many clinical implications when discussing stroke and inflammation. Stroke is one of the leading causes of death worldwide, with few available treatments that aid in long-term recovery. Ischemic stroke, which accounts for roughly 80% of all stroke incidence, is caused by a blockage that reduces blood flow and oxygen to the brain. In comparison, hemorrhagic strokes are caused by a ruptured artery or vessel that subsequently enables RBCs to enter the brain, leading to oxidative stress. When looking specifically at a hemorrhagic stroke, free heme- and hemoglobin (Hb)-induced neuroinflammation and cell death been have shown to worsen patient outcomes and exacerbate symptoms the longer they are left untreated [14]. One potential theory to improve outcomes is to reduce free-circulating RBCs, the haptoglobin and hemoglobin (Hp–Hb) complex, and the heme–hemopexin complex, which, respectively, bind to CD36, CD163, and LRP1 [15,16,17]. The soluble forms of these receptors are proposed to act as binding proteins to these molecules, although with slightly less affinity. The mechanism of activity and clearance will be explored later on in the discussion. A diagram of receptor binding is illustrated in Figure 2 to clarify each molecule’s interactions with its corresponding decoy receptor.

In addition to reported predictive abilities, soluble receptors have been shown to function as immunomodulators, to enhance tumor apoptosis, have anti-angiogenic effects, and exert anti-inflammatory function [18,19]. Moreover, soluble receptors are being explored to prevent toxicity in the membrane receptor [20]. Given this, discerning the pharmacodynamics of soluble receptors has revolutionized medical sciences, and soluble receptors have been implemented as therapeutic targets in several disease conditions [21]. Pharmacodynamically, soluble receptors can act as agonists or antagonists [5]. For example, in granulocyte-macrophage colony-stimulating factor (GM-CSF), soluble receptors may function as competitive antagonists with the membrane-associated receptor for the ligand [22]. This is consistent with earlier models, suggesting that soluble receptors function as binding proteins and prevent a ligand’s breakdown, which can be seen in receptor growth hormone [23].

After accounting for the numerous functions of soluble receptors, this review intends to examine the role of important soluble receptors, such as sCD36, sLRP1, and sCD163. Each of these three receptors is involved in pathways that help scavenge immunoglobin and heme and are of particular interest when discussing stroke and subsequent outcomes [24]. These receptors’ specific roles in stroke and related neuroinflammation are reviewed, topics that have remained esoteric and understudied. Here, we aim to synthesize the available research on this topic to help researchers battling time constraints to advance their work.

## 2. sCD36 Receptor

CD36 is a multifunctional, transmembrane, glycoprotein scavenger receptor belonging to the class B family receptors originally found in platelets [11,25]. CD36 is encoded on chromosome 7 in humans and consists of a single peptide chain of 472 amino acids with a molecular weight of 88 kDa [26,27]. Structurally, CD36 has two intracellular domains, a large extracellular domain, and two transmembrane domains [28]. The presence of 10 *N*-linked glycosylation sites in the extracellular domain causes extensive variability in the molecular mass and contributes to the diversity of ligand binding, discussed later [15,29,30,31]. The intracellular domain, on the other hand, comprises two very short cytoplasmic tails with pairs of palmitoylated cysteines, which aids in the adherence of CD36 to the membrane [32]. The extensive structure of CD36 contributes to its various binding associations, including native lipoproteins (high-density lipoprotein (HDL), low-density lipoprotein (LDL), and very-low-density lipoprotein (VLDL)), oxidized LDL, long-chain fatty acids, advanced glycation end products (AGEs), thrombospondin, and apoptotic neutrophils [33,34,35]. Because increases in LDL and AGE are associated with acute ischemic stroke incidence, CD36 is considered a particularly important tool when evaluating effective targets for the regulation of stroke outcomes [36,37].

In addition to CD36′s structure contributing to its ligand diversity, the CD36 receptor can be found on diverse cell types such as adipocytes, monocytes, epithelial cells, platelets, and skeletal muscle cells [38]. This diversity has also revealed the numerous and complex roles CD36 plays in the human body. Several of these include biological functions related to the immune system, monocyte and macrophage metabolism, the phagocytosis of apoptotic cells, the activation of transforming growth factor β, fatty acid regulation, and the transmembrane transportation of oxidized LDL. Other roles include acting as an adhesion molecule and platelet activator as well as functioning in angiogenesis. It has even been noted that the CD36 receptor causes differentiation from monocytes to macrophages in response to inflammatory and proatherogenic mediators [25,39]. Therefore, it is evident that CD36 may play a part in protecting against oxidative damage resulting from hemorrhage due to its role in coagulation and the regulation of oxidized LDL transport.

On the other hand, CD36 exhibits associations with known predisposing factors to stroke, emphasizing its complex and dynamic function. For example, CD36 is reportedly present during the initiation and progression of atherosclerosis by internalizing LDL and forming lipid-filled macrophage foam cells [40]. Moreover, one study revealed that CD36 might promote vascular amyloid deposition and cerebrovascular damage, eventually leading to cognitive impairment and neurovascular dysfunction [41]. This would pose a significant potential risk for hemorrhagic stroke. Adding to the complexity of the CD36 receptor, opposing results have been published indicating that CD36 can both facilitate and inhibit long-chain fatty acid transport [28,42]. The involvement of CD36 in various biological pathways emphasizes this molecule’s pharmacologic potential and, subsequently, its soluble form.

The soluble form of CD36 in plasma is known as sCD36 or the CD36 receptor ectodomain [43,44]. Contrary to prior studies, it has been reported that sCD36 exists as microparticles, which are unbound proteins or peptides liberated from cells such as adipocytes, platelets, and macrophages following a physiological stimulus [11,13,45]. The reported pleiotropic action of CD36 contributes to the versatility of its corresponding soluble receptor. For example, studies have shown that sCD36 is a marker of macrophage activation, inflammation, and increased fat accumulation in the vessel wall and may exhibit varied expression due to circadian regulation with atherosclerosis [46,47,48]. Additionally, sCD36 has been identified as a biomarker for various pathological conditions, such as type 2 diabetes mellitus, metabolic syndrome, obesity, cardiovascular diseases (e.g., atherosclerosis), and plaque stability [13,45,46,49,50,51]. In addition to the cerebrovascular effects, each of the previously listed conditions are known precursors to stroke and, therefore, may implicate sCD36 as a potential therapeutic target when combating stroke incidence.

### 2.1. sCD36 and Hemorrhagic Stroke

CD36 has been shown to play protective and regulative roles in both intracerebral hemorrhages (ICH) and subarachnoid hemorrhages (SAH). During a hemorrhagic stroke, the BBB is compromised, leading to an increased presence of CD36 in the brain. One study reports that CD36 promotes hematoma absorption after ICH and that this clearance is positively correlated with improved patient outcomes when compared to CD36-deficient groups [52]. In addition, one preclinical model revealed similar results, reporting that CD36^−/−^ mice had increased neuronal injury and detrimental outcomes compared to wildtype (WT) mice after SAH [53]. Due to CD36′s ability to bind thrombospondin and initiate platelet coagulation, it is evident that this receptor plays a crucial role in erythrocyte regulation and clearance after a hemorrhagic stroke. Therefore, there is a foundation for continued preclinical research into the soluble form of CD36 and the dynamic functions it plays in alleviating detrimental stroke outcomes. For example, it is worth investigating the circulation potential of sCD36 to promote coagulation and its access to the brain following ICH or SAH.

### 2.2. sCD36 and Ischemic Stroke

Compared to hemorrhagic stroke, CD36 plays a significantly different role in the pathophysiology following an ischemic stroke. While it was previously mentioned that CD36 is a target receptor for oxidized LDL and AGEs, it is important to note that these molecules are also released following an ischemic stroke. The upregulation of these molecules reportedly plays a destructive role in stroke outcomes, including vascular dementia and muscle weakness, due to the formation of signaling complexes with toll-like receptors (TLR) and the subsequent development of atherosclerosis [54,55]. Moreover, another study revealed that CD36 upregulation in hyperlipidemic subjects considerably aggravated patient outcomes after ischemic stroke. For instance, it was reported that CD36 might promote foam cell formation in macrophages after ischemic injury, significantly contributing to inflammation and atherosclerosis progression [56]. During plaque destabilization, throughout the development of thrombotic formation, it is reported that sCD36 is markedly increased, and there is also a succeeding escalation of acute ischemic events [50]. Considering the soluble form of CD36, an increase in circulating pro-inflammatory protein levels can increase stroke incidence and substantially exacerbate outcomes. Therefore, the dynamic capabilities of sCD36 require additional insights and should be closely monitored in a clinical setting.

## 3. sLRP1 Receptor

The LRP1 receptor is a large, multifunctional, scavenger type I transmembrane endocytic receptor that functions in cell signaling. The LRP1 receptor is also known as the cluster of the differentiation 91 receptor (CD91). LRP1 belongs to a gene family found across many species, including Drosophila, Xenopus mammals, and *C. elegans* [57]. This genetic conservation across phyla may indicate its evolutionary and physiological importance.

The LRP1 gene encodes and synthesizes the two mature chain forms from a single chain weighing 600 kDa after proteolytic processing [57]. The LRP1 receptor is divided into three parts: an ectodomain, a transmembrane domain, and the intracellular tail. The extracellular portion functions in ligand binding and consists of the heavy α chain weighing 515 kDa. The heavy chain is noncovalently bound to the transmembrane light β-chain, which weighs 85 kDa. The intracellular tail contains the serine and tyrosine phosphorylation sites, which undergo phosphorylation and execute the signal transduction process [58]. The presence of the YXXL motif and distal dileucine repeat in the structure of LRP1 is predominantly responsible for LRP1 endocytosis [59].

LRP1 binds to approximately 40 ligands, including tPA, the hemopexin–heme complexes, amyloid precursor protein (APP), Aβ wildtype and mutant peptides, growth factors, apolipoprotein E (ApoE), α-2-macroglobulin (α-2M), proteinase inhibitors (plasminogen activator inhibitor-1), factor VIII, protein S, bacteria, viral proteins, and lactoferrin [60]. In addition, LRP1 primarily functions as a cargo transporter due to its rapid endocytotic rate. The cytoplasmic domain interacts with adaptor proteins, such as disabled-1, Shc, c-Jun amino-terminal kinase–interacting protein, FE65, and post-synaptic density protein 95 (PSD-95), which suggests the role of LRP1 in the signal-transducing receptor. Thus, LRP1 exhibits a dual role as a rapid cargo transporter and transmembrane cell-signaling receptor [58].

The LRP1 receptor is shed from the cell by metalloproteinases ADAM10, ADAM17, and MMP14. Structurally, the β-chain is truncated, while the α-chain is intact and can be detected in the plasma, cerebrospinal fluid (CSF), peripheral nervous system, and brain [61]. This truncated portion of the actual receptor is the soluble form of the receptor, accomplished through the process of ectodomain shedding [62]. The cleavage of the β-chain of LRP by beta-secretase (BACE) also causes the release of sLRP1 into the extracellular fluid [63]. After being released from the cell membrane, the soluble receptor has an affinity for LRP1 ligands in the extracellular space, including tPA and potentially heme–hemopexin complexes, but it loses the ability to move the ligands into the cell by transcytosis [62]. By competing with membrane-bound LRP1 for its ligands, sLRP1 potentially plays an important role in heme scavenging, BBB permeability, and inflammation and pathophysiological processes that are important in stroke.

### 3.1. sLRP1 Scavenges Heme after Hemorrhagic Stroke

After a hemorrhagic stroke, excess erythrocytes in the brain release hemoglobin, which, when degraded, releases free heme into the brain [60]. Free heme and its oxidized form, hemin, contribute to inflammatory damage and cell death after stroke by ferroptosis or necroptotic signaling [64]. The sLRP1 receptor may bind to the heme–hemopexin complex and therefore reduce the amount of bioactive heme in the brain after stroke [65]. Suppose sLRP1 has the ability to scavenge inflammatory blood products such as heme–hemopexin from the brain after a stroke. In that case, it could improve stroke outcomes by reducing inflammation, neuronal death, and edema caused by excess heme.

### 3.2. sLRP1 Regulates Blood–Brain Barrier Permeability through tPA after Stroke

The BBB permeability plays an important role in the pathophysiology of both ischemic and hemorrhagic stroke [60]. Cerebral ischemia causes increased BBB permeability, which leads to edema and brain damage [66]. Polavarapu et al. found that, during cerebral ischemia, tPA induces LRP1 shedding from astrocytes, which increases the permeability of the BBB and results in cerebral edema and brain damage [67]. sLRP1 also appears to be implicated in hemorrhagic transformation. Su et al. demonstrated that administering tPA during ischemic stroke compromises BBB permeability and causes ICH [68]. It is evident that sLRP1 plays a role in stroke outcomes through the regulation of BBB permeability.

### 3.3. sLRP1 Regulates Neuroinflammation after Stroke

In addition to roles in heme scavenging and BBB permeability, sLRP1 is also involved in neuroinflammation, which plays an important role in stroke pathogenesis. Cerebral ischemia results in cellular damage and causes extravascular tissue to release inflammatory mediators [69]. LRP1 is expressed at the site of inflammation on microglia, the resident immune cells of the brain, and is involved in microglial activation. Brifault et al. conducted a study on transgenic LysM^Cre^-positive-LRP1^fl/fl^ and LysM^Cre^-negative-LRP1^fl/fl^ mice to study the role of sLRP1 on neuroinflammation [61]. The LRP1 ligand receptor-associated protein (RAP) increased microglial activation and the release of pro-inflammatory mediators, such as TNF, interleukin-1β (IL-1β), and interleukin-6 (IL-6), and the effect was attenuated with LRP1-deficient cells. The study also showed that sLRP1 shedding was increased by lipopolysaccharide (LPS) and RAP in a 24-h period, starting from 6 h. In wildtype microglia treated with purified sLRP1 at 60 ng/mL in vitro in a 0.5% serum-supplemented medium, the concentration of TNF mRNA, ILβ mRNA, and IL-6 mRNA increased significantly in a concentration-dependent manner (*p* < 0.05, *p* < 0.01, and *p* < 0.001, respectively). The ability of sLRP1 to increase cytokine production in LRP1-expressing and LRP1-deficient microglia in LysM*^Cre^*-positive-LRP1^fl/fl^ and LysM*^Cre^*-negative-LRP1^fl/fl^ mice was not statistically significant. sLRP1 amplified the microglial response to RAP in immunoblot analysis. Preventing LRP1 shedding inhibits the induction of pro-inflammatory mediators by RAP (*p* < 0.001) [61]. Based on this study’s results, sLRP1 and LRP1 play a key role in neuroinflammation by regulating cytokine expression and cell signaling in microglia, implicating sLRP1 in many disease processes in which microglia release pro-inflammatory mediators, including stroke.

While sLRP1 plays a potentially neuroprotective role in stroke by possibly acting as a scavenger for heme-hemopexin complexes that are found in excess in the brain after a hemorrhagic stroke, LRP1 shedding increases the permeability of the BBB during ischemic stroke, leading to cerebral edema, tissue damage, and, in some cases, hemorrhagic transformation. In addition, LRP1 shedding causes the release of inflammatory cytokines from microglia during neuroinflammation. Because sLRP1 appears to display both anti-inflammatory and pro-inflammatory activity during various disease processes, further research needs to be conducted on the role of sLRP1 in hemorrhagic and ischemic stroke before clinical recommendations can be made.

## 4. sCD163 Receptor

CD163, also identified as M130, is a 130-kDa membrane protein expressed predominantly on the surface of cells of the monocyte/macrophage lineage and is noted to be in the highest abundance on alternatively activated macrophages (M2). CD163 serves as a unique class B scavenger receptor with a short cytoplasmic tail, a single transmembrane segment, and an ectodomain with nine cysteine-rich scavenger receptor domains; most notably acting as a Hb scavenging receptor [70,71,72].

Concerning the systemic expression of CD163, the two predominant phenotypes of macrophages should be identified. The activated “M1” branch of macrophages is triggered by the products of Th1 lymphocyte and natural killer cells, including IFNγ, IL-12, and IL-18 [72]. These forms of macrophages generally perpetuate a pro-inflammatory cellular signaling pathway. Conversely, the alternatively activated macrophages, historically so-called M2, are activated by IL-4 and IL-13 and typically contribute to anti-inflammatory signaling [72]. Furthermore, some studies have indicated the presence of another macrophage phenotype, termed Mhem, which carries a high iron load, heme oxygenase 1 (HO1) activity, and CD163 expression compared to both M1 and M2. Notably, Mhem has been localized to hemorrhages in atherosclerotic plaques and has been identified as having an anti-inflammatory role. Interestingly, Mhem has been theorized to arise from the conversion of present M1. The dynamic nature of macrophage function and protein expression plays an important role when analyzing the function of CD163. Signaling molecules were shown to upregulate the macrophage expression of CD163, including glucocorticoid, IL-6, IL-10, and Hb, whereas IL-4, IFNγ, LPS, TNF, CXCL4, and GM-CSF have been shown to downregulate it [71]. Once CD163 is expressed on the surface, the macrophage becomes implicated in a dynamic process of mediating inflammatory and oxidative conditions involved in stroke via serum Hb regulation [73].

CD163 confers downstream anti-inflammatory capability as an Hb scavenger receptor by initiating a cascade of reactions to induce HO1 activity and through the secretion of anti-inflammatory heme metabolites. Specifically, CD163 binds to Hb complexed to Hp with high affinity, which is then transported into the macrophage via receptor-mediated endocytosis. It is also worth noting that CD163 binds to free Hb with low affinity, particularly when Hp has been saturated with Hb after severe hemolysis. Upon binding the Hp–Hb complex, the CD163 receptor cross-links and the Hp–Hb complex are endocytosed and degraded within a lysosome by HO1 to three primary anti-inflammatory products: Fe^2+^, carbon monoxide (CO), and biliverdin, which is further broken down to bilirubin. Furthermore, this process is accompanied by the release of IL-6 and GM-CSF [71,74]. The modulatory nature of CD163 in the inflammatory cascade makes it a great candidate for studying the potential point of mediation in stroke damage.

It has also been reported that membrane-bound macrophage CD163 binds TWEAK in a dose-dependent manner. Some believe this implies that CD163 prevents TWEAK from enacting its normal physiological signaling. In contrast, others point toward TWEAK interaction with sCD163 as part of a cascade leading to tissue repair and erythropoiesis via erythrocyte interaction. Ultimately, the medical implication has not yet been elucidated [75,76,77]. As the macrophage-bound CD163 receptor’s various functions become apparent, we turn our attention to the soluble form’s dynamic role, also known as a decoy, receptor form of CD163, i.e., sCD163.

The extracellular domain of the CD163 receptor is shed from activated macrophages by proteolytic cleavage through the activation of metalloproteinases, thereby increasing the sCD163 plasma levels [78]. sCD163 shares roughly 94% of the extracellular part of the membrane-bound CD163 (mCD163), which constitutes nine scavenger receptor cysteine-rich domains [79]. This cleavage removes the ligand-binding site from scavenger domain 3 and, afterward, enables the cross-linking of CD163. Furthermore, the region in CD163 domain 3 is sensitive to proteolytic degradation in vitro [80]. This protease-induced shedding of CD163 has also been observed experimentally using phorbol myristate acetate and inhibited by protein kinase C inhibitors [81,82].

Studies point to ADAM17, also known as TACE, to be the enzyme most responsible for cleaving the sCD163 form from macrophages. Similarly, ADAM10 is also responsible for the cleavage of CD163 to the soluble form [8]. This process has been hypothesized to occur in pathological states of increased inflammation, such as in the presence of LPS, leading to increased serum TNF and sCD163 [71]. Furthermore, research from previous groups points toward observing sCD163 levels as a prognostic marker for inflammation [8,71]. Therefore, in attempting to both analyze and mitigate inflammation during and following stroke conditions, CSF-derived sCD163 becomes a strong candidate for future study. However, systemic variables leading to increased or decreased levels of sCD163 vary widely, and additional research must be conducted to evaluate the role of sCD163 as a biomarker and its functionality in stroke pathology.

Although the specific function of sCD163 is unclear at this point, Etzerodt et al. have made strides in distinguishing two separate soluble forms of the receptor and their potential physiological relevance. It is hypothesized that 10% of healthy patients’ sCD163 is membrane-bound on extracellular vesicles (EVs) instead of soluble ectodomain CD163, which is the product of traditional cleavage methods. The authors of this study concluded that there should be a recognized designation between EV-CD163 and ectodomain CD163, as they noted differential distributions of serum-soluble CD163 in sepsis cases and exposures to endotoxin. They reported a more robust increase in EV-CD163 in patients with sepsis than a more substantial increase in ectodomain CD163 in exposure to endotoxin; thus, the authors recommend continued research to differentiate the two. For the purposes of discussion in this paper, “sCD163” encompasses both of these forms unless noted otherwise [77]. Furthermore, the existence of these two separate forms designates a need for future research of clinical conditions where each is distinctly produced.

Typical systemic values for sCD163 have not reached a clinical standard; however, one study reports a mean concentration in the CSF of 53.2 ng/mL with a CSF/serum quotient seven times that of albumin [83]. This suggests that sCD163 production is localized to the CNS.

### 4.1. sCD163, the Immune System and Stroke

As mentioned previously, sCD163 has been proven to be intricately involved in immune function. With the immune system’s role being pivotal to stroke outcomes, it is essential to analyze the ways inflammation-related physiology involving sCD163 can impact stroke and subsequent outcomes.

Studies have revealed serum levels of sCD163 as a marker of macrophage activity and as inversely related to mCD163 [84]. The sCD163 receptor also has anti-inflammatory activity and exclusively exhibits a direct inhibitory effect on T cell proliferation [85,86]. Thus, Etzerodt et al. mentioned that the rise in sCD163 could be a prognostic marker in inflammation [71]. The dynamics between the sCD163 and CD163 receptors from the macrophages suggest the magnitude of inflammation [87]. The levels of CD163 rise during the resolution phase of inflammation.

sCD163 also exhibits immunomodulatory actions. For example, it inhibits phorbol ester (a phlogistic agent)-induced lymphocytic growth, can cause macrophage activation and proliferation when present at elevated levels, and, most notably, can inhibit the production of activated T lymphocytes [8,88,89,90]. Therefore, with sCD163 being a receptor for the pro-inflammatory and pro-oxidant Hb bound to Hp, and the role immune cells play in the secondary damage of both ischemic and hemorrhagic stroke, further research on the relevant pathways for pharmaceutical modulation must be studied [71,91].

### 4.2. sCD163 and Hemorrhagic Stroke

With secondary toxic hemolytic products, pro-inflammatory molecules, and oxidative damage resulting from ICH in both the short and long term, research has begun to point toward sCD163 to indicate hemorrhagic stroke severity as well as a point of modulation.

Current research has shown that sCD163 is a viable candidate to indicate hemorrhagic stroke occurrence and a quantifiable marker for severity [73,83,92]. It has been well identified in animal models that CD163 is induced in microglia and neurons after Hb exposure [93]. However, this measure is not applicable in a clinical setting, making sCD163 an enhanced analysis subject regarding neuronal Hb exposure. The serum and CSF levels of sCD163 were analyzed in patients who experienced ICH. This study concluded that increased intrathecally derived sCD163 receptors in the serum were associated with increased hematoma and perihematomal edema (PHE) volume. Furthermore, patients with larger PHE expansion showed worse modified Rankin Scale (mRS) scores. Although the mechanism sCD163 played in ICH’s pathophysiology is unclear, the authors suggest that this result is likely due to sCD163 detoxification and resolution of the hematoma leading to reduced secondary injury [73].

Similarly, the reported effect of an increased level of sCD163 has also been noted in the CSF of patients (n=30) suffering from SAH. However, in contradiction to the aforementioned ICH study, the authors of this study note that the increased sCD163 comes as a by-product of increased localization of CD163(+) macrophages to the CNS after SAH rather than independently controlled increased shedding of CD163. The separation between these two processes can be denoted by whether Hp–Hb scavenging is increased or impeded. It is hypothesized that increased CSF sCD163 resulting from the influx of CD163 into CSF followed by shedding results in lower CSF Hp compared to increased shedding, which interferes with Hp–Hb scavenging [83,94]. The presence of increased levels of macrophages expressing CD163 after SAH has been confirmed; however, whether this is directly translated to increased sCD163 or the function of sCD163, once cleaved, has not been elucidated [92].

The inconsistent nature of this research re-emphasizes the argument that additional experimental testing needs to be completed. Impactful clinical observations have been made; however, we are left with sizable gaps in knowledge regarding the background mechanism at this point time.

### 4.3. sCD163 and Ischemic Stroke

While the acute immune response has been reported to be beneficial to ischemic stroke prognosis, it has been well documented that prolonged a pro-inflammatory milieu will lead to excessive secondary neuroinflammation and poor prognosis [95]. To mitigate the propagation of pro-inflammatory molecules, the immune attenuation capacity of sCD163 should be brought to the forefront [8,88,89].

The sCD163, thus far, has been hypothesized to improve stroke outcomes via the sequestration of Hb via interaction with the Hp–Hb complex. Thereby sCD163, combined with microglia-bound CD163, would reduce the amount of free Hb, minimizing associated pro-inflammatory and pro-oxidant reactions [80,96,97].

Experimental studies of sCD163 as a prognostic marker of stroke and a potential modulation site have been scarce, although promising. Hypothesized by authors to be a driving component in the termination of inflammation and possible reduction in autoimmune damage, sCD163 was studied in a group of 39 patients who had suffered an ischemic stroke compared to 20 controls and 20 stroke mimics [98]. Blood analysis concluded that ADAM17 activity was increased in patients suffering from ischemic stroke, corroborating an increase in analyzed levels of sCD163 in patients from multiple studies [98,99]. Furthermore, sCD163 levels were negatively associated with lymphocyte activity after a stroke in patients, as previously mentioned. Notably, a compound variable, indexing ADAM17 mRNA expression, CD163 mRNA expression, and cellular TACE activity was correlated with worse National Institute of Health Stroke Severity (NIHSS) scores. This study and others analyzing sCD163 as a biomarker highlight the necessity for continued research in the mediation of the process of shedding sCD163 [97,98,100].

In sum, there is a gap in the current understanding regarding the mechanism behind increased production of sCD163 as well as its function in inflammatory attenuation. Promising literature has verified that quantifying sCD163 can be a viable predictor of neurological functionality, with the greatest current limitation being small-to-moderate sampled patient populations. Further elucidating the functionality of sCD163 with refined clinical and preclinical research is a necessity as inflammation and other Hb-related secondary processes become a focus of stroke treatment.

## 5. Future Considerations

When reviewing soluble receptors in terms of different types of stroke, there is limited availability of published research that focuses on their unique interactions and the potential therapeutic benefits, despite the abundance of research of soluble receptors in various other diseases. Even with said research, a pharmacotherapeutic agent has yet to be discovered that selectively targets the soluble receptors with desired bioavailability features, minimal drug interaction and drug incompatibility, and flexibility in the route of administration. More research needs to be conducted regarding the possibility and efficacy of directly administering soluble receptor analogs as a potential therapy in order to limit the free levels of these respective toxic ligands. Research is currently exploring the roles of various ligands for soluble receptors, the effectiveness of targeted therapy, and demographic variables affecting receptor interactions and using cutting-edge technology to rapidly identify protein interactions. One purpose of identifying important protein–protein interactions is regulating and maintaining oxidative stress and endothelial dysfunction that are key risk factors for stroke. By targeting soluble receptors and elucidating their interactions (and modulating the bioavailability and clearance of toxic ligands), it is possible to mitigate harmful outcomes and promote recovery post-stroke.

Recently, decoy nanoparticles’ interactions with the membrane-bound receptor have been studied and revealed the ability of soluble receptors to block specific ligand connections with the cell-surface receptor [101]. This could be of particular importance when looking at the potential blockage of oxidized LDL to its receptor and the subsequent removal of circulating LDL as a therapeutic strategy against atherosclerosis development. The targeted prevention of atherosclerosis via the occlusion of LDL is a major development toward lowering stroke incidence and prevalence, particularly ischemic stroke. Other ligand interactions are also being explored to understand their roles in neuroinflammation and soluble receptor shedding. For example, LPS and calreticulin reportedly stimulate LRP1 release, which leads to the downstream expression of pro-inflammatory cytokines and subsequent neuroinflammation [61]. Targeting specific ligands can inhibit their downstream processes and may play an important role in stroke prevention and disease regulation.

Research is looking into using a treatment therapy known as the antibody-decoy strategy. This is a targeted therapy where both the soluble receptor and the native receptor antibody target the ligand and the receptor, respectively. This therapy has been successfully used in preclinical models to hinder tumor development and invasion and should be considered a therapeutic strategy in various stroke models [102]. Demographic variables, such as age, sex, disease stages, and genetics are also evaluated to determine the relationship between soluble receptors and disease pathology [103]. Understanding and uncovering the underlying mechanisms of these aspects will help generate new therapeutic targets with the potential to prevent the initial onset or development of stroke and other diseases and mitigate harmful side effects and outcomes.

Presently, improved protein–protein interaction modulators could be synthesized. The use of sophisticated drug discovery tools, such as automated quantitative high-throughput screening (qHTS) assay, time-resolved Förster resonance energy transfer, and bead-based luminescent oxygen-channeling assay formats (AlphaScreen^®^) has enabled us to generate superior drug candidates. The use of computational models is expanding, allowing us to quantify soluble receptors’ mechanisms and more accurately evaluate these receptors as diagnostic or prognostic biomarkers [77,104]. Using recombinant DNA technology, drugs are being developed with more affinity for the targets and with minimal side effects. Thus, our goals should be to generate a soluble receptor with structural modification that can target as many relevant molecules, limiting the bioavailability and improving clearance of the toxic ligands that aggravate the disease process [105]. When specifically looking at the various types of stroke, receptors such as sCD36, sLRP1, and sCD163 should be evaluated as potential therapeutic targets due to the vast diversity in their ligand associations and reported targets, notably in regard to the intrinsic oxidative stress and proinflammation properties of the RBC, the hemoglobin, and the heme.

The soluble receptors’ role as a biomarker is also being evaluated in various conditions, either as a new or an additional selective marker. In some instances, it has been documented that those soluble receptors, as with other biomarkers, can discriminate several disease conditions and can be a useful tool against that disease [106]. Implementing cutting-edge technology, such as FirePlex^®^ particle technology and bead-based immunoassays for flow cytometers, antibody microarray, and Luminex^®^ technology will enable fast and easier responses in the future to detect multiple proteins at one time. The measurement of both the membrane and soluble form of the receptor is essential to label it as a suitable biomarker [81]. Therefore, analytical considerations should be made in the future, specifically regarding circulating soluble receptors in terms of in vitro stability, biological variations, reference ranges, and comparisons to diseased cohorts.

In addition, detailed mechanistic studies need to be conducted to understand the simultaneous shedding of soluble and membrane receptors, as well as to uncover the methods that limit this process. It is critical to determine the biological significance of soluble receptor pathways that pose as potential pharmacotherapeutic agents in neurological disorders, as well as prognostic biomarkers and therapeutic targets. Future therapies should focus on the upregulation of soluble receptors in stroke and other neurological conditions where concentration is relevant [107].

Moving forward, it is also recommended that researchers use larger sample sizes and maintain rigid protocols in a uniform method of collection at established time intervals to prevent confounding bias. Additionally, follow-up longitudinal studies should be conducted in reference to the existing studies to confirm accurate data reporting. Similarly, acute studies should be followed by chronic studies. Promising preclinical studies should be carried forward to study the correlation with long-term functional recovery, as they may provide relevant diagnostic and prognostic translations in human studies. In unifying efforts to understand the underlying mechanisms that soluble receptors play in different stroke models, we can make substantial strides toward the prevention and elimination of a myriad of illnesses.

## 6. Study Design and Limitations

Information for this scoping review was gathered using search engine tools such as PubMed, Google Scholar, and University of Florida library database, OneSearch. The specific strategy consisted of a variety of searches with the basic pattern of ““Injury” AND “Soluble Receptor”” where the queried injuries included ICH, SAH, ischemic stroke, and the soluble receptors included sCD36, sCD163, sCD91, sLRP1. All variants of shorthand and longhand notation for each respective term was used in these searches. While this search strategy encompassed the existing data relevant to this topic, as a scoping review as opposed to a systematic review, there may be data missing from this manuscript. Additionally, with some journals being reluctant to publish insignificant data, there may be a lack of available opposing data. We acknowledge there may be shortcomings in the data collected and presented in this manuscript but hope that this review will propagate the further exploration of this topic.

## Abbreviations

AD	Alzheimer’s disease
ADAM	A disentegrin and metalloproteinase
AGEs	Advanced glycation end products
ANI	Asymptomatic neurocognitive impairment
ApoE	Apolipoprotein
APP	Amyloid precursor protein
BACE	Beta-site amyloid precursor protein cleaving enzyme 1 or beta secretase
BBB	Blood-brain barrier
CD36	Cluster differentiation 36
CD163	Cluster differentiation 163
CNS	Central nervous system
CSF	Cerebrospinal fluid
HO1	Heme oxygenase-1
Hp	Haptoglobin
ICH	Intracerebral hemorrhage
IFNγ	Interferon gamma
IL	Interleukin
LDL	Low density lipoprotein
LRP1	Low-density lipoprotein receptor-related protein 1
MMPs	Matrix metalloproteinases
MND	Mild neurocognitive disorder
mRS	Modified Rankin score
PHE	Perihematomal edema
RAGE	Receptor for advanced glycation end products
RBC	Red blood cell
sRAGE	Soluble receptor for advanced glycation end products
ROS	Reactive oxygen species
SAH	Subarachnoid hemorrhage
TACE	TNF-converting enzyme
TBI	Traumatic brain injury
TBSA	Total body surface area
TLR	Toll-like receptors
TNF	Tumor necrosis factor
tPA	Tissue plasminogen activator
TWEAK	Tumor necrosis factor-related weak inducer of apoptosis
WBC	White blood cell
WT-LRPIV	Wildtype LRP cluster IV
